# Synergistic Effects of Ag Nanoparticles/BiV_1-x_Mo_x_O_4_ with Enhanced Photocatalytic Activity

**DOI:** 10.1186/s11671-017-2345-9

**Published:** 2017-11-09

**Authors:** Mengting Yu, Shixiong Zhou, Qingguo Meng, Haiqin Lv, Zhihong Chen, Yongguang Zhang, Mingliang Jin, Mingzhe Yuan, Xin Wang, Guofu Zhou

**Affiliations:** 1 South China Academy of Advanced Optoelectronics, South China Normal University, Guangzhou, Guangdong Province China; 2 Shenyang Institute of Automation, Chinese Academy of Sciences, Guangzhou, 511458 China; 3 International Academy of Optoelectronics at Zhaoqing, South China Normal University, Guangzhou, Guangdong Province China

**Keywords:** Hydrothermal synthesis, Photocatalytic, Metal doping, Ag/BiV_1-x_Mo_x_O_4_

## Abstract

In recent years, BiVO_4_ has drawn much attention as a novel photocatalyst given its excellent ability to absorb visible light. This work reports the development of Ag-modified BiV_1-x_Mo_x_O_4_ composites through a facile hydrothermal synthesis with the subsequent photoinduced reduction of Ag^+^ at almost neutral pH conditions. Metallic Ag nanoparticles were deposited on the (040) facet of Mo-doped BiVO_4_ powders. The crystal structure and morphology of the as-prepared samples were studied by XRD and SEM analyses. Moreover, the photocatalytic performance of BiVO_4_, Ag/BiVO_4_, and Ag-modified BiV_1-x_Mo_x_O_4_ were evaluated by the degradation of rhodamine B (RhB). The Ag/BiV_0.9925_Mo_0.0075_O_4_ composite exhibited the most efficient photocatalytic performance. The present work provides greater insight into the application of BiVO_4_ in the field of photocatalysis.

## Background

Given the increasing environmental pollution and energy crises, the development of efficient and promising solutions to reduce energy shortages and protect the environment is paramount [1, 2]. Photocatalyst-based semiconductors, such as Bi_2_WO_6_ [3, 4], BiPO_4_ [5, 6], Ag_3_PO_4_ [7, 8], and BiVO_4_ [9–13], have attracted much attention due to their applications in the degradation of organic pollutants or hydrogen production from water splitting. Nevertheless, most of the existing oxide photocatalysts have very low light-response efficiencies primarily because they only respond to ultraviolet light due to their narrow bandgaps [14–16]. Additionally, the photoinduced electrons can easily recombine with holes leading to a lower optical performance [17, 18].

Due to its visible photocatalytic activity, wide bandgap of 2.42 eV, high stability, and non-toxicity, bismuth vanadate (BiVO_4_) is a promising n-type semiconductor photocatalyst [19–21]. However, its resulting carrier transfer efficiency is relatively poor, leading to the recombination of photogenerated electrons and holes, which severely limits the photocatalytic performance of BiVO_4_. Various studies have assessed BiVO_4_ modifications [20, 22–24], and substitution or metal doping on BiVO_4_ has been shown as the most efficient method to change its carrier transport efficiency. Metal element doping introduces new defects or charges in the crystal lattice [25], influencing the motion of electrons and the creation of holes under light irradiation [26, 27]. Adjustments to the distribution status or changes in the band structures can lead to changes in the activity of semiconductors [28]. For example, Thalluri et al. [29] introduced hexavalent molybdenum (Mo) at an almost neutral pH to substitute V while preserving the atomic ratio of fBiVO_4_, leading to the formation of a good crystal structure and considerable photocatalytic activity for water oxidation. Mo has a higher valence than V and therefore strengthens the n-type characteristics of the material [30]. Additionally, the photocatalytic activity of BiVO_4_ is highly dependent on its various crystal facets. Recent studies on the deposition of noble metals, such as Ag, Cu, and Au, on the different facets of BiVO_4_ have demonstrated good photocatalytic activity [31–33]. Li et al. [34] produced an Ag/BiVO_4_ composite through the hydrothermal synthesis and photoreduction of Ag deposited on the (040) crystal facets of BiVO_4_, leading to an enhanced photoelectrochemical performance, as indicated by the fast separation of the electron–hole pairs.

In the present study, we build on the facile hydrothermal synthesis approach of Li et al. [29] to obtain BiV_0.9925_Mo_0.0075_O_4_ in weakly alkaline conditions, coupled with photoreduction deposition of Ag nanoparticles on the (040) facets of the as-produced substrate materials. Ag/BiV_0.9925_Mo_0.0075_O_4_ composite photocatalysts were successfully synthesized and showed enhanced photocatalytic degradation of rhodamine B (RhB) under xenon lamp irradiation (*λ* > 420 nm) compared to the non-composite Ag-deposited or Mo-doped BiVO_4_ materials. Herein, we report the preparation, characterization, and photocatalytic activity of BiVO_4_, Ag/BiVO_4_, BiV_1-x_Mo_x_O_4_, and Ag/BiV_1-x_Mo_x_O_4_ composites.

## Experimental

### Synthesis of BiVO_4_ and BiV_1-x_Mo_x_O_4_ Powders

Bismuth nitrate pentahydrate (Bi(NO_3_)_3_·5H_2_O, analytical grade), ammonium metavanadate (NH_4_VO_3_, analytical grade), ammonium carbonate, and ammonium molybdate ((NH_4_)_2_MoO_4_) were obtained from Sigma–Aldrich and used as received, without any further purification. All other chemicals used in the experiments were also of analytical grade, and deionized water was used for the preparation of the solutions. In a typical process, 3.7 mmol of Bi(NO_3_)_3_·5H_2_O, 3.7 mmol of NH_4_VO_3_, and 12 mmol of (NH_4_)_2_CO_3_ were dissolved in 75 mL of 1 M HNO_3_ and stirred for approximately 30 min at room temperature until a clear solution was obtained. The pH of the mixture was adjusted to pH 8 with NaOH (2 M). The mixture was transferred into a 150-mL Teflon-lined stainless autoclave and heated for 12 h at 180 °C under autogenous pressure in an oven. The precipitate was filtered and washed three times with distilled water followed by ethanol and dried for 12 h at 60 °C in a drying oven.

The doped samples were prepared by replacing the equivalent weight of NH_4_VO_3_ with different amounts of Mo. Mo precursors were introduced such that a nominal 0.5, 0.75, and 1% atomic substitution of V was achieved.

### Preparation of Ag/BiVO_4_ and Ag/BiV_1-x_Mo_x_O_4_ Samples

BiVO_4_ (0.50 g) and AgNO_3_ (0.05 g) were added to a (NH_4_)_2_C_2_O_4_ (0.8 g L^−1^, 100 mL) aqueous solution in a 250-mL beaker in an ultrasonic bath until an evenly dispersed solution was formed. The resulting yellow mixture was then irradiated with a Xenon lamp for 30 min under magnetic stirring. The color of the system turned from a vivid yellow to grayish-green, indicating the generation of metallic Ag in the reaction system. The resulting samples were then filtered, washed with DI water, and dried at 60 °C for 12 h to obtain the Ag/BiVO_4_ and Ag/BiV_1-x_Mo_x_O_4_ composites.

### Photocatalytic Activity

Assessment of the photocatalytic activity was performed using the degradation rate of RhB. The experimental system for photodegradation was calibrated at a UV cut-off wavelength below 420 nm, and the irradiation height of the Xenon lamp was close to the height of the 250-mL beaker. In a typical procedure, the as-prepared photocatalyst (0.1 g) was well dispersed in a RhB aqueous solution (150 mL, 10 mg L^−1^) under ultrasonication in a glass reactor equipped with a cooling water circulator to maintain a reaction system temperature of room temperature. The suspension was stirred for 30 min in the dark to reach the adsorption–desorption equilibrium and was then irradiated for 2 h with a Xenon lamp (300 W) under continuous stirring. A 5-mL aliquot of the suspension was taken every 30 min and centrifuged. The absorption spectrum of the obtained liquid supernatant was measured in reference to the absorption intensity of RhB at 552 nm.

### Characterization Techniques

The morphologies of the pure BiVO_4_ and the decorated composites were investigated by field emission scanning electron microscopy (FESEM, S4800) and transmission electron microscopy (TEM; JEM-2100F, JEOL). Elemental analysis was performed by X-ray photoelectron spectroscopy (XPS; VGESCA-LAB MKII) with a non-monochromatic Mg Kα X-ray source. The crystalline phase of the samples was determined by X-ray diffraction (XRD; Bruker D8) with Cu Kα radiation. Inductively coupled plasma (ICP) was employed to analyze the elemental composition of the samples. Additionally, UV–vis diffuse reflectance spectrum measurements were performed using a Shimadzu spectrophotometer (UV-2450) to evaluate the bandgap energy of BiVO_4_, Ag/BiVO_4_, BiV_1-x_Mo_x_O_4_, and Ag/BiV_1-x_Mo_x_O_4_ over a wavelength range of 360–800 nm.

## Results and Discussion

**Fig. 1:**
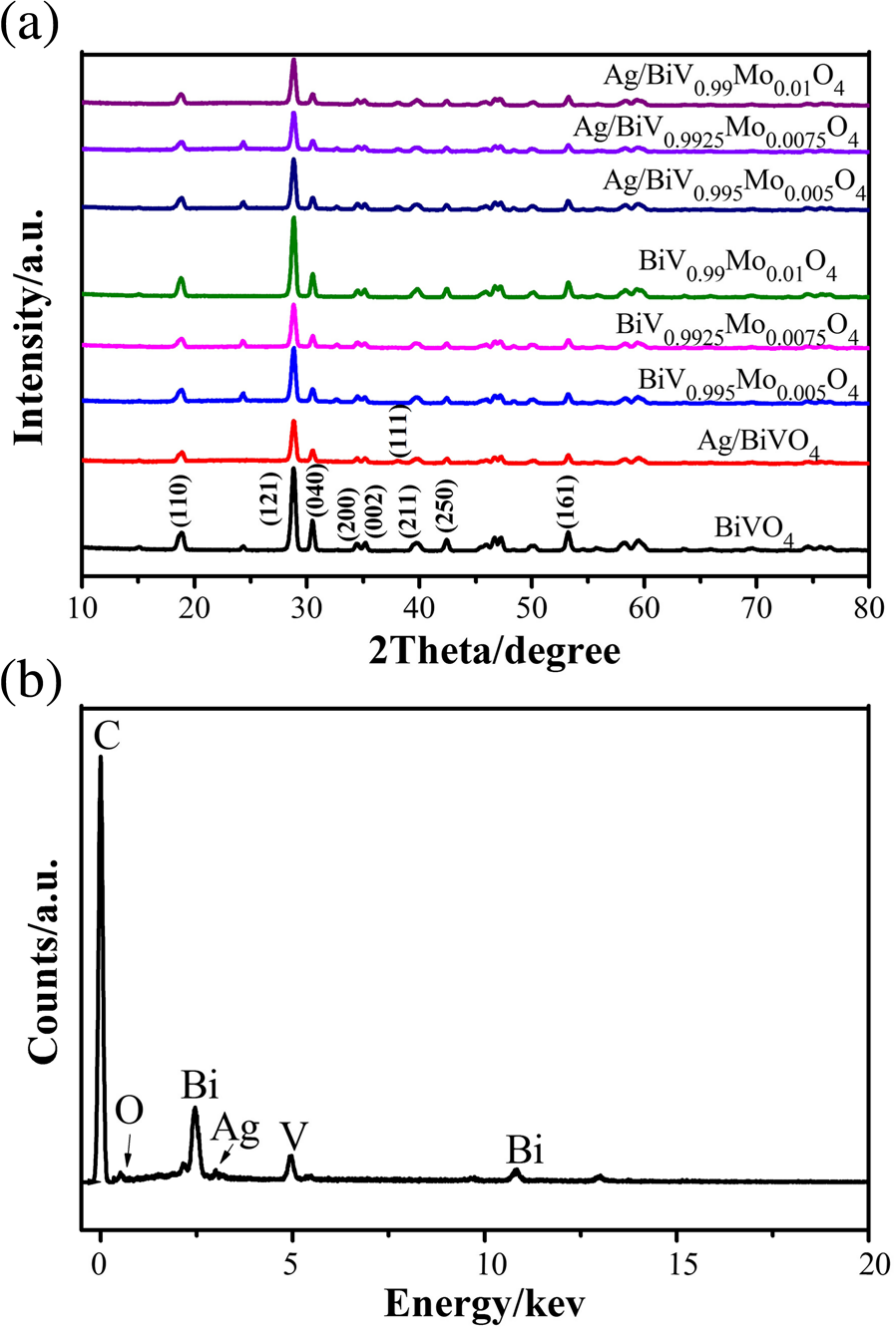
**a** XRD patterns of pure BiVO_4_, Ag/BiVO_4_, BiV_1-x_Mo_x_O_4_, and Ag/BiV_1-x_Mo_x_O_4_. **b** The corresponding EDX analysis of Ag /BiV_0.9925_Mo_0.0075_O_4_

The crystallographic structure and phase of the prepared composites were characterized by XRD analysis (Fig. 1a). The sharp diffraction peaks observed in the as-prepared BiVO4 were assigned to the conventional BiVO_4_ phase since they were in good agreement with the standard (JCPDS) card no. 14-0688. According to the peak splitting observed at 18.7° and 30.5°, which indicate the (110) and (040) facets, the prepared BiVO_4_ material possessed a single monoclinic scheelite structure. A diffraction peak at 38.1° was observed in the Ag-related photocatalysts (Fig. 1a) corresponding to the (111) crystal phase of metallic Ag (JCPDS file: 65-2871). This indicates that the photoreduction of Ag^+^ ions indeed occurred, leading to the deposition of Ag nanoparticles on the BiVO_4_ and BiV_1-x_Mo_x_O_4_ surfaces. Nevertheless, due to the low relative content of Ag, the XRD peaks were not intense.

**Fig. 2:**
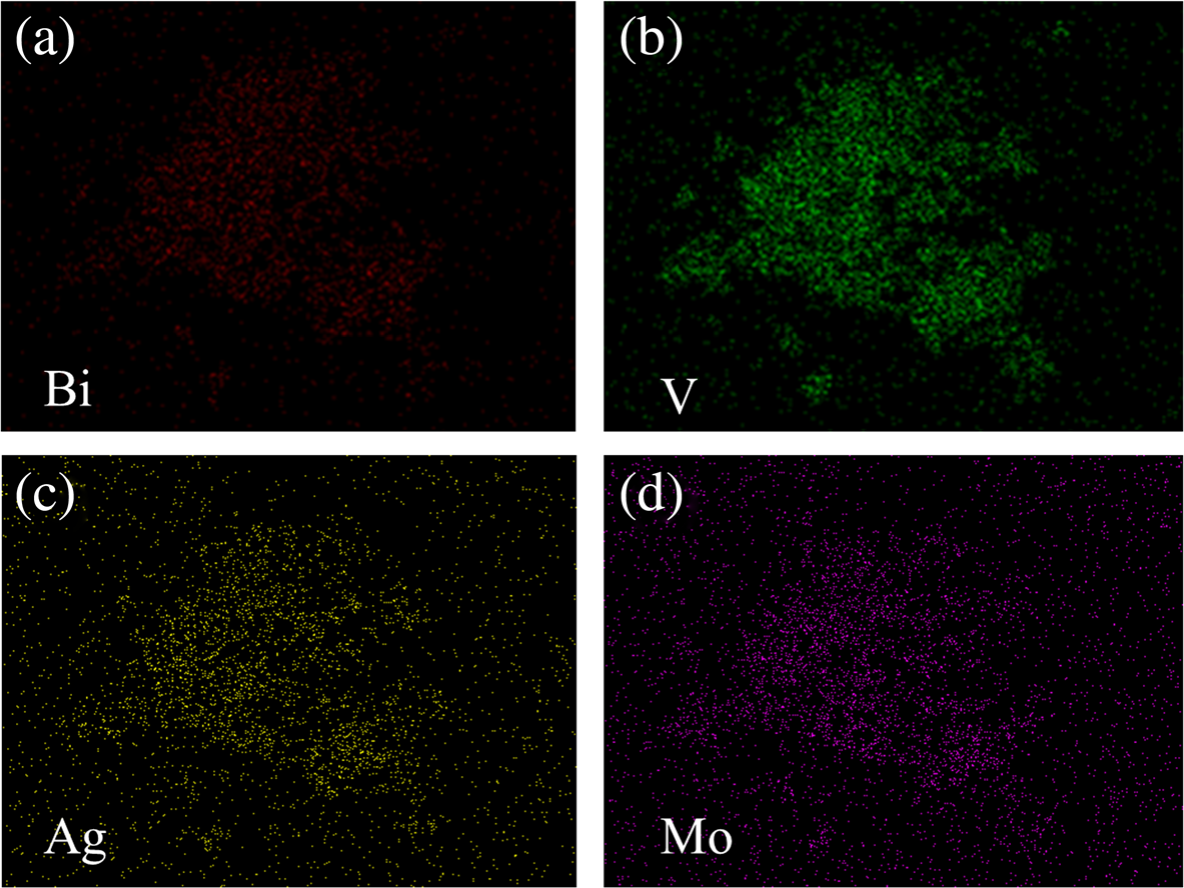
**a**–**d** The corresponding elemental mapping analysis of Bi, V, Ag, and Mo in Ag/BiV_0.9925_Mo_0.0075_O_4_, respectively

As shown in Fig. 2a, EDS confirmed the presence of the Ag species, which agrees with the XRD results. The Bi (Fig. 2b), O (Fig. 2c), V (Fig. 2d), Mo (Fig. 2e), and Ag (Fig. 2f) elements are all distributed uniformly in the Ag/BiV_1-x_Mo_x_O_4_ composites, and the results verify the existence of Mo and Ag. The relative amounts of Mo did not appear to affect the crystal structure or phase. The Mo substitution ratio was assessed by ICP (Table 1); the practical Mo atomic content was calculated to be 0.16% in Ag/BiV_0.9925_Mo_0.0075_O_4_. It was observed that, although the nominal dopant content introduced with the precursors was 0.75%, the final resulting amount of Mo in the doped materials was always lower than the expected. Similar results have also been found in previous research, and it is possible that intrinsic losses and the evaporation of the Mo dopant occur during the hydrothermal synthesis processes [35, 36].

**Table 1:** Properties of the pure BiVO_4_, Ag/BiVO_4_, BiV_0.9925_Mo_0.0075_O_4_, and Ag/BiV_0.9925_Mo_0.0075_O_4_ powders

Sample	BiVO_4_	Ag/BiVO_4_	BiV_0.9925_Mo_0.0075_O_4_	Ag/BiV_0.9925_Mo_0.0075_O_4_
Bandgap (eV)	2.30	1.61	2.18	1.78
Atomic% of Ag dopant from ICP	–	6.28446	–	5.92476
Atomic% of Mo dopant from ICP	–	–	0.163704	0.167735
Atomic% of Ag dopant from XPS	–	6.03	–	4.72
Degradation rate (%)	6.4	8.4	9.6	97.9

**Fig. 3:**
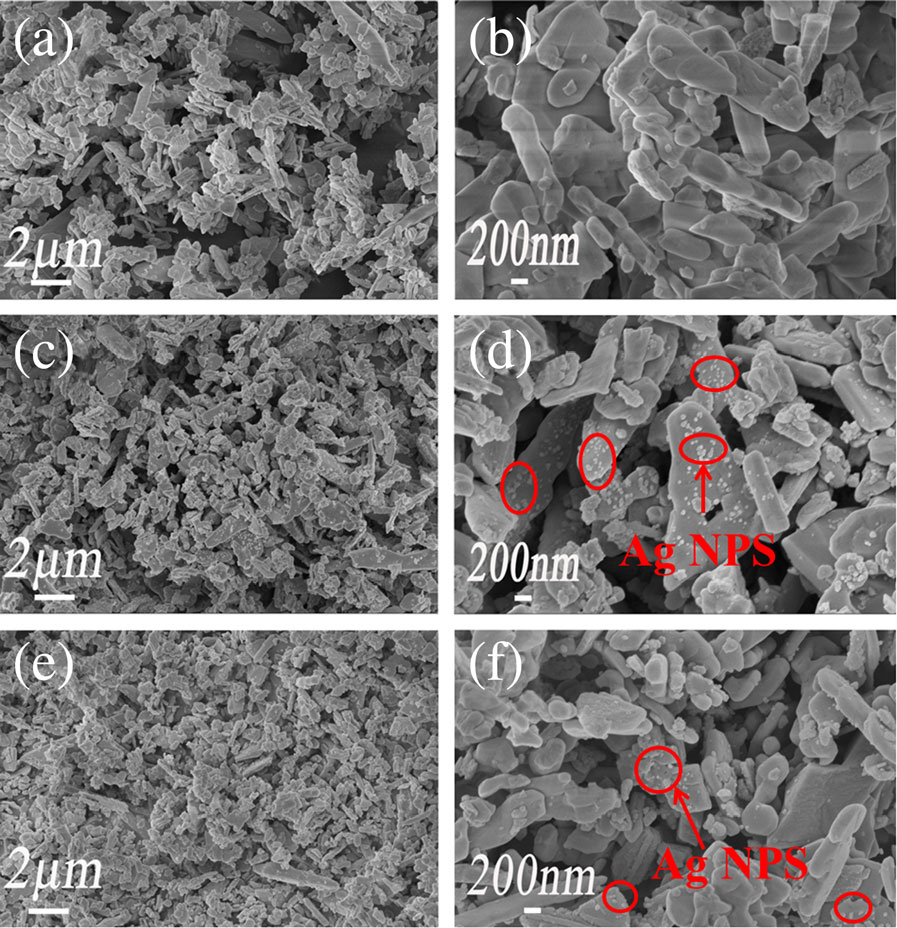
SEM images. **a**, **b** Low- and high-magnification images of pure BiVO. **c**, **d** Low- and high-magnification images of the Ag/BiVO_4_ composite. **e**, **f** Low- and high-magnification images of the Ag/BiV_0.9925_Mo_0.0075_O_4_

**Fig. 4:**
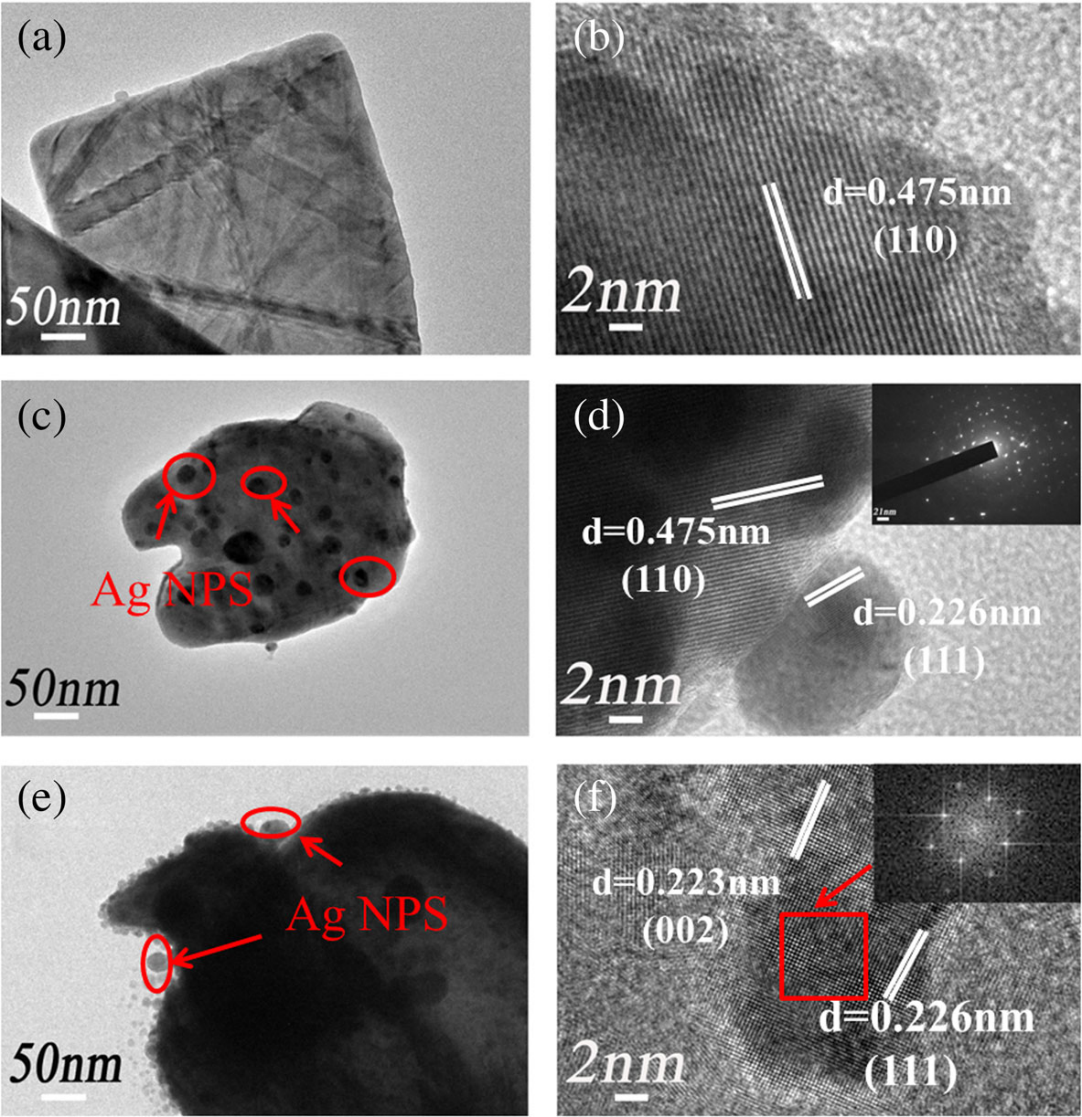
TEM images of **a** pure BiVO_4_, **c** Ag/BiVO_4_, and **e** Ag/BiV_0.9925_Mo_0.0075_O_4_ and **b**, **d**, and **f** high-magnification images of **a**, **c**, and **e**, respectively

The morphology of the as-prepared pure BiVO_4_, Ag/BiVO_4_, and Ag/BiV_1-x_Mo_x_O_4_ were investigated by SEM (Fig. 3). Pure BiVO_4_ showed a slice-layer morphology with several clusters (Fig. 3a, b). For Ag/BiVO_4_, metallic Ag was observed to be well dispersed on the (040) crystal facet (Fig. 3c), which agrees with the XRD analysis. The images of Ag/BiV_0.9925_Mo_0.0075_O_4_ composite at different magnification were shown in Fig. 3e, d. Uniformly shaped metallic Ag nanoparticles were clearly observed on the surface of Ag/BiV_0.9925_Mo_0.0075_O_4_ (Fig. 3d) likely due to the high exposure of the (040) surface. This crystal facet has been shown to have a good charge carrier mobility [37]. Thus, the observed morphology should be beneficial to the photocatalytic performance of the synthesized doped BiVO_4_ powders. The as-prepared BiVO_4_, Ag/BiVO_4_, and Ag/BiV_0.9925_Mo_0.0075_O_4_ samples were further observed by TEM (Fig. 4a). Interplanar spacings of 0.475 nm were clearly observed in Fig. 4b, corresponding to the (110) crystallographic facet of BiVO_4_ (JCPDS Card No. 14-0688). The crystal lattice fringe at 0.226 nm belonged to the (111) plane of metallic Ag nanoparticles in the Ag/BiVO_4_ and Ag/BiV_0.9925_Mo_0.0075_O_4_ samples (Fig. 4d, f). Based on the above analyses, metallic Ag was successfully deposited onto the BiV_0.9925_Mo_0.0075_O_4_ surface, leading to a good connection between Ag and the Mo-doped BiVO_4_ and promoting effective electron and hole separation in the composite system.

**Fig. 5:**
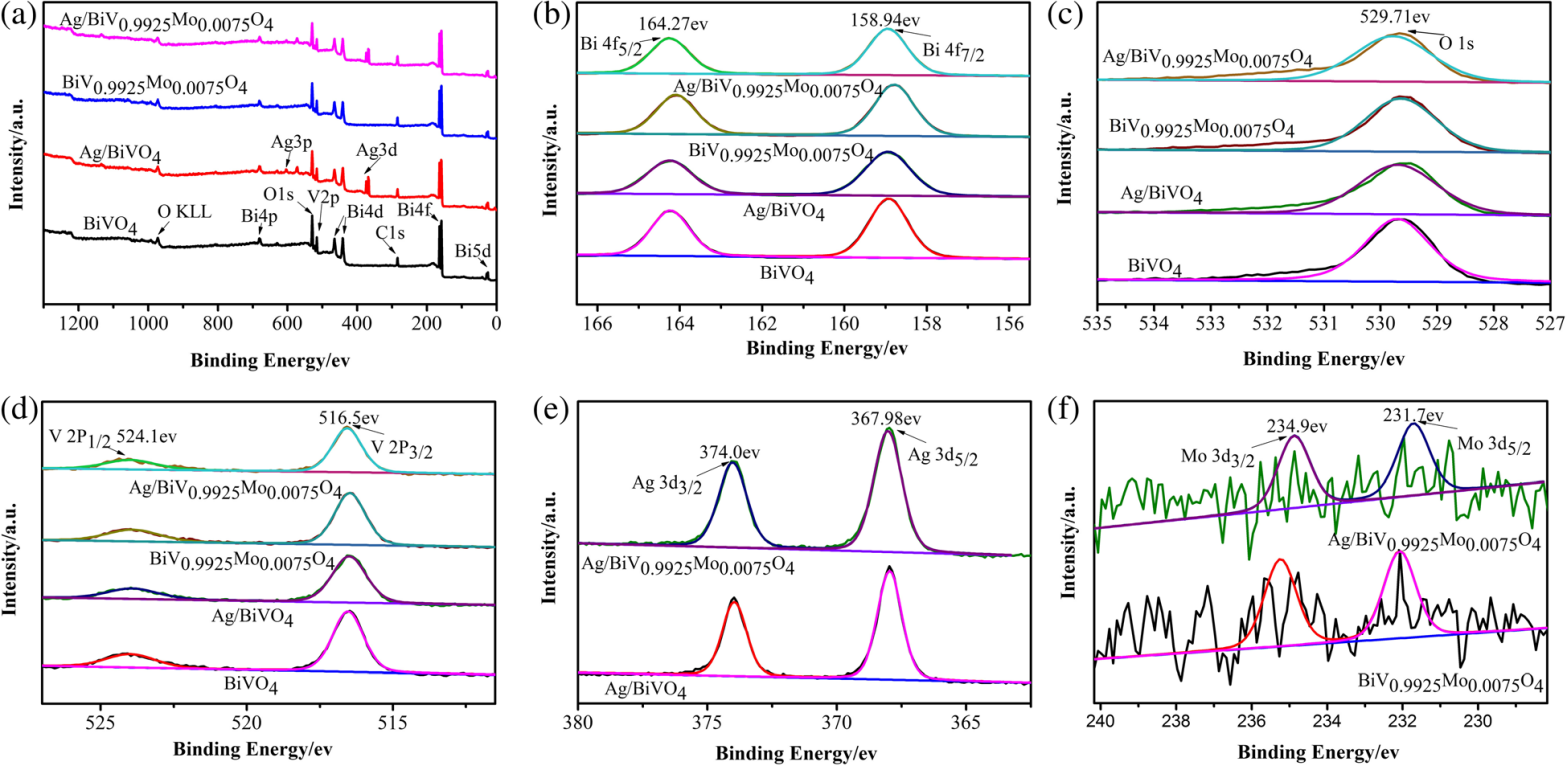
XPS spectra of the as-prepared photocatalyst. **a** The survey XPS spectrum, **b** Bi 4f, **c** O 1s, **d** V 2p, **e** Ag 3d, and **f** Mo 3d peaks related to the photocatalyst

XPS analysis of the as-prepared samples confirmed the presence of Bi, V, O, Ag, and Mo (Fig. 5a). The binding energies of Bi 4f were 158.94 and 164.27 eV, corresponding to Bi 4f^7/2^ and 4f^5/2^, respectively, confirming the Bi^3+^ peaks in BiVO_4_ (Fig. 5b). A typical O 1s spectrum was observed, as indicated by the main characteristic peak at 529.71 eV (Fig. 5c). The V 2p^3/2^ and 2p^1/2^ peaks observed at 516.5 and 524.1 eV, respectively, indicated the existence of V^5+^ (Fig. 5d). The Ag 3d peaks at 367.98 and 374.0 eV, corresponding to Ag 3d^5/2^ and 3d^3/2^ (Fig. 5e), respectively, were observed in both Ag/BiVO_4_ and Ag/BiV_0.9925_Mo_0.0075_O_4_, confirming the existence of the metallic Ag species. Furthermore, the molar ratio of metallic Ag species accounted for 6.6% of all elements, as determined by XPS and in agreement with the ICP measurements (Table 1). Finally, the Mo 3d^5/2^ and 3d^3/2^ peaks located at 231.7 and 234.9 eV (Fig. 5f), respectively, confirm the presence of Mo^6+^.

**Fig. 6:**
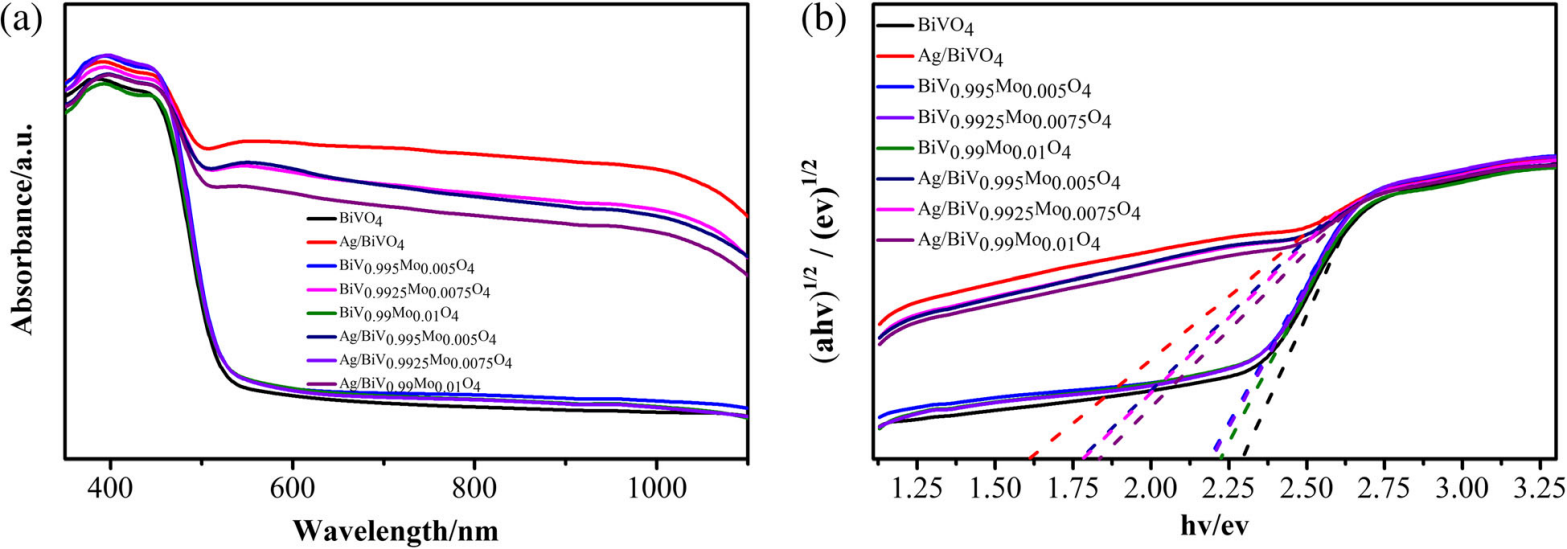
The photophysical properties of the as-prepared materials. **a** UV–vis diffuse reflectance spectra of the BiVO_4_, Ag/BiVO_4_, BiV_1-x_Mo_x_O_4_, and Ag /BiV_1-x_Mo_x_O_4_. **b** Energy bandgap evaluation of the corresponding materials

UV–vis diffuse reflectance spectrum measurements were taken to evaluate the optical bandgap and absorption properties of the photocatalysts, as shown in Fig. 5. The photocatalytic activity of a semiconductor is largely dependent on the size of the bandgap; the narrower the bandgap is, the greater the shift is of the absorption wavelength towards longer wavelengths. The bandgap of as-prepared BiVO_4_ was approximately 2.3 eV (Fig. 6b), which agrees with the Kubelka–Munk bandgap estimation theory [38]. Compared with BiVO_4_, all the Mo-doped samples showed relatively narrow bandgaps (Fig. 6b). Furthermore, all Ag-deposited BiVO_4_ and BiV_1-x_Mo_x_O_4_ photocatalysts exhibited strong absorption in the visible light range in Fig. 6a. The Ag/BiVO_4_ photocatalyst exhibited the best light absorption. The absorbance of as-prepared Ag/BiV_0.9925_Mo_0.0075_O_4_ was between that of BiVO_4_ and Ag/BiVO_4_, thus indicating that the introduction of Mo hindered the photoresponsive characteristics of Ag. However, it is worth pointing out that, in addition to photoabsorption, other characteristics can also significantly influence the photocatalytic efficiency of photocatalysts.

**Fig. 7:**
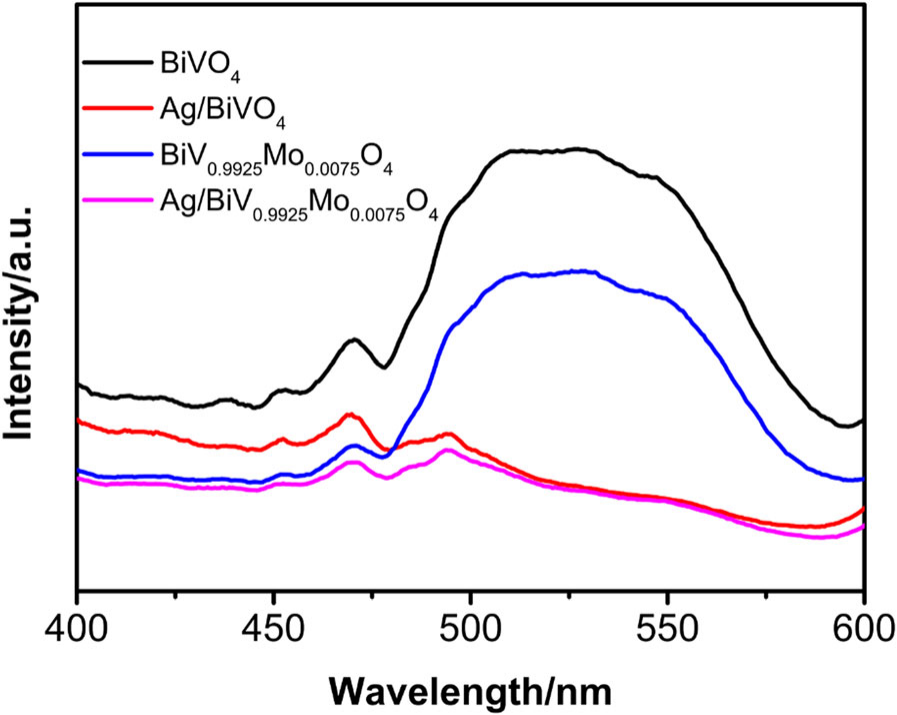
Photoluminescence spectra of pristine BiVO_4_, Ag/BiVO_4_, BiV_0.9925_Mo_0.0075_O_4_, and Ag/BiV_0.9925_Mo_0.0075_O_4_ composites

Photoluminescence (PL) spectras were taken to investigate the separation efficiency of the photogenerated electron–hole pairs. The PL spectra of pure BiVO_4_, BiV_0.9925_Mo_0.0075_O_4_, Ag/BiVO_4_, and Ag/BiV_0.9925_Mo_0.0075_O_4_ composites, with an excitation wavelength of 310 nm, are shown in Fig. 7. BiVO_4_ and BiV_0.9925_Mo_0.0075_O_4_ show a prominent emission band centered at approximately 510 nm. The order of the intensity of the PL spectra was BiVO_4_ > BiV_0.9925_Mo_0.0075_O_4_ > Ag/BiVO_4_ > Ag/BiV_0.9925_Mo_0.0075_O_4_. Because a lower PL intensity indicates a higher separation efficiency, this would lead to a higher photocatalytic activity in the overall system. Consequently, the higher photocatalytic performance of Ag/BiV_0.9925_Mo_0.0075_O_4_ agrees with the PL measurement.

**Fig. 8:**
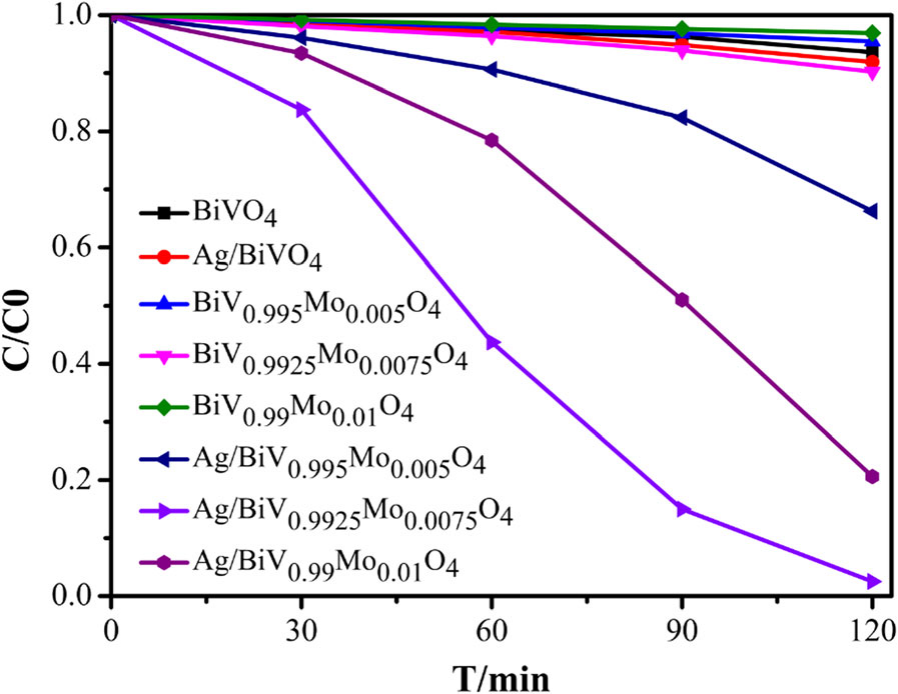
Photocatalytic degradation of RhB by BiVO_4_, Ag/BiVO_4_, BiV_1-x_Mo_x_O_4_, and Ag/BiV_1-x_Mo_x_O_4_ photocatalysts

The photocatalytic decomposition results, according to the degradation of RhB under visible light (*λ* > 420 nm), confirmed Ag or Mo alone had little effect on the catalytic activity of BiVO_4_ under light irradiation for 2 h (Fig. 8). Conversely, the deposition of Ag on Mo-doped BiVO_4_ showed effective photocatalytic activity, with the variation of the Mo content, showing a difference in photocatalytic activity. Ag/BiV_0.9925_Mo_0.0075_O_4_ exhibited an extremely efficient degradation of RhB under visible light irradiation with full decolorization after 2 h while only 7, 8, and 10% degradation was achieved over BiVO_4_, Ag/BiVO_4_, and BiV_0.9925_Mo_0.0075_O_4_, respectively. Thus, Mo-doped Ag-deposited BiVO_4_ was able to suppress the charge recombination and greatly enhance the efficiency of the photocatalytic process.

**Fig. 9:**
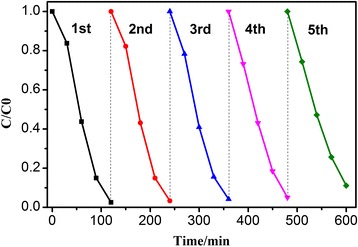
Five cycle runs of Ag/BiV_0.9925_Mo_0.0075_O_4_ for the photodegradation of RhB under visible light irradiation

The stability and reusability of photocatalysts are very important for their practical application. Therefore, we assessed the repeated cycles of Ag/BiV_0.9925_Mo_0.0075_O_4_ in the photocatalytic degradation of RhB for 2 h under visible light irradiation. Overall, 99% of the RhB solution was degraded after five cycles (Fig. 9), indicating that the sample exhibited good photocatalytic stability.

**Fig. 10:**
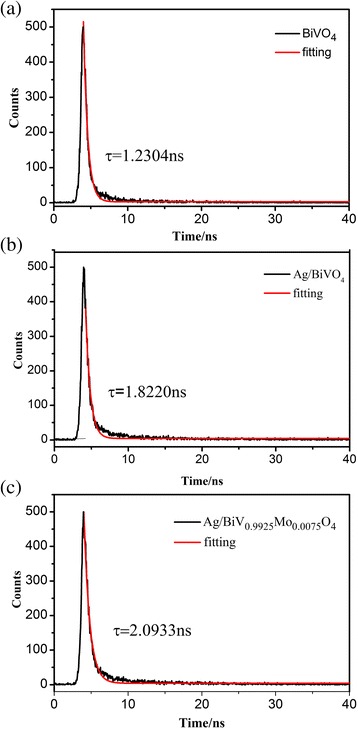
Ns-level time-resolved fluorescence decay curves of as-prepared **a** BiVO_4_, **b** Ag/BiVO_4_, and **c** Ag/BiV_0.9925_Mo_0.0075_O_4_ composite

To further assess the separation efficiency, the charge carrier lifetimes of pure BiVO_4_, Ag/BiVO_4_, and Ag/BiV_0.9925_Mo_0.0075_O_4_ were also analyzed (Fig. 10). The decay curves for the as-prepared photocatalysts fit well to a double-exponential function. The charge carrier decay lifetimes of BiVO_4_, Ag/BiVO_4_, and Ag/BiV_0.9925_Mo_0.0075_O_4_ composites were 1.2304, 1.8220, and 2.0933 ns, respectively. Thus, the Ag-deposited samples, both with and without Mo doping, had much longer charge carrier lifetimes than pure BiVO_4_, achieving effective photocarrier separation and suggesting that a synergistic effect among Ag, Mo, and BiVO_4_ led to enhancements of the photocatalytic activity.

**Fig. 11:**
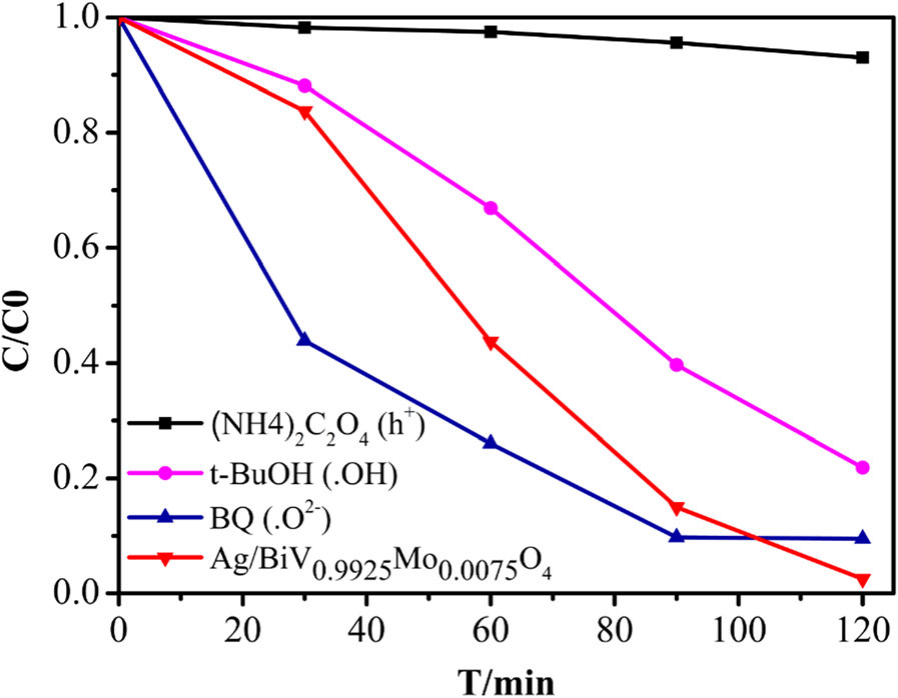
Plots of photogenerated carrier trapping in the system during the photodegradation of RhB by Ag/BiV_0.9925_Mo_0.0075_O_4_

To explore the underlying photocatalytic mechanism, RhB degradation was conducted under visible light irradiation [39], adding a hole (h^+^) scavenger (ammonium oxalate ((NH_4_)_2_C_2_O_4_)), a superoxide radical (•O^2−^) scavenger (1.4-benzoquinone, BQ) [40], or hydroxyl radical (•OH) scavengers (tert-Butanol, t-BuOH) [41]. Following the addition of BQ, no obvious decrease was observed, but an acceleration in the degradation rate was detected compared to that of Ag/BiV_0.9925_Mo_0.0075_O_4_ (Fig. 11). The faster degradation rate may have resulted from the SPR-effect of metallic Ag in Ag/BiV_0.9925_Mo_0.0075_O_4_, which would enhance the separation efficiency of electrons and holes. However, when t-BuOH was added, the catalytic efficiency decreased from 97.5 to 78.1%, indicating the presence of •OH as the active species. The photocatalytic activity was drastically reduced with the addition of (NH_4_)_2_C_2_O_4_, suggesting that the holes acted as the main active species.

**Fig. 12:**
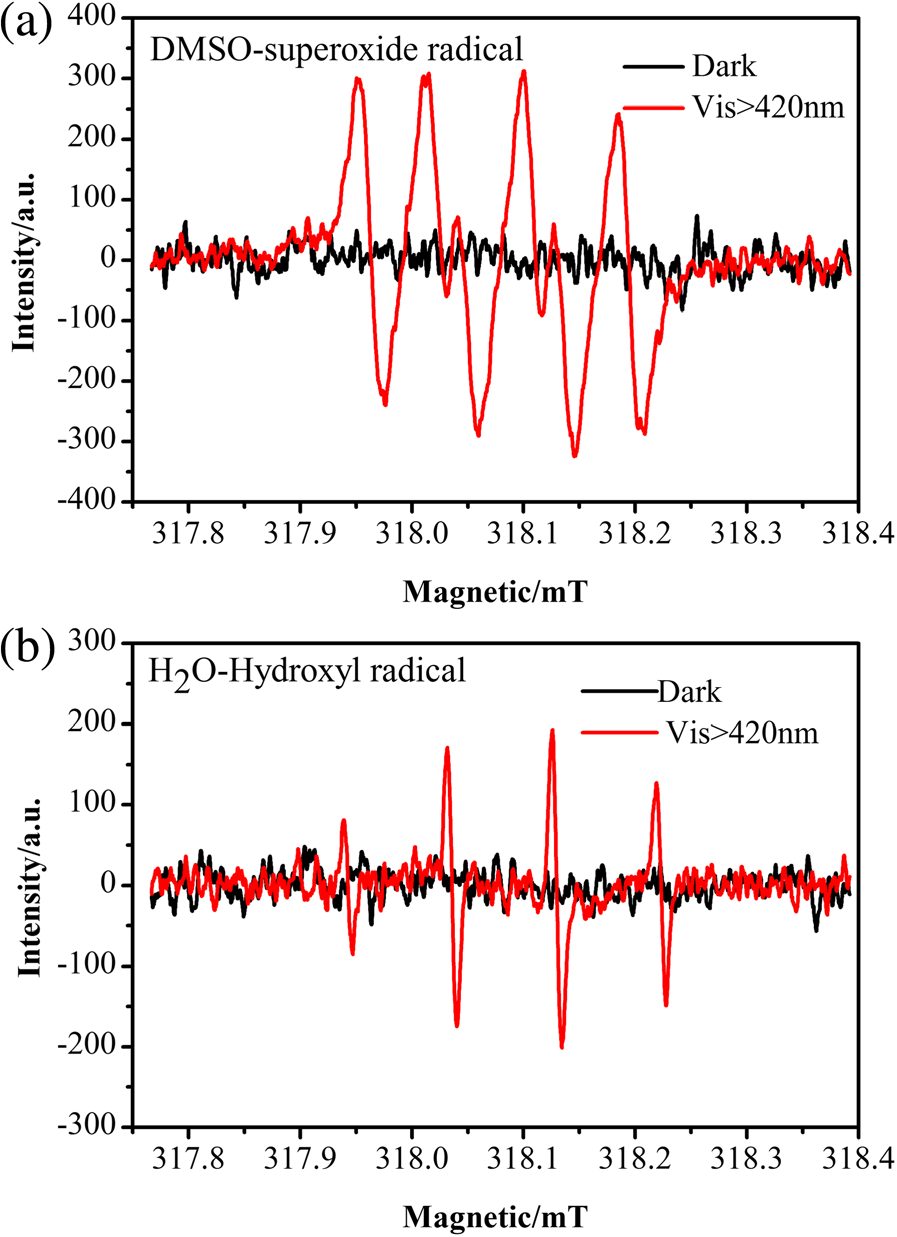
Electron paramagnetic resonance (ESR) spectra of Ag/BiV_0.9925_Mo_0.0075_O_4_ in **a** DMSO solvents and **b** water

To further confirm the main active species generated in the photocatalytic process, electron spin resonance (ESR) was used. The principle of ESR is to react with free radicals using a spin-trapping agent to generate a relatively stable free radical adduct. A peak intensity was observed under visible light compared with dark conditions (Fig. 12a), demonstrating the existence of •O^2−^. In addition, obvious signals (Fig. 12b) suggested that •OH was produced in the photocatalytic process. In conclusion, the radical trap experiments and ESR analysis revealed that the photocatalytic process was governed by the combined effect of h^+^, •O^2−^, and •OH active species.

**Fig. 13:**
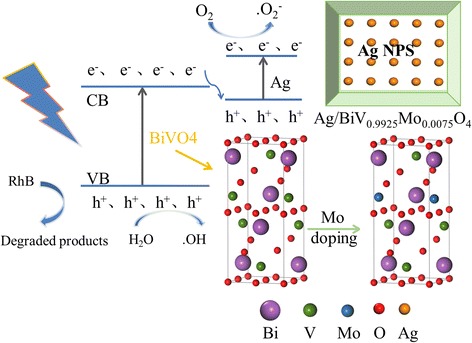
Schematic mechanism of charge transfer in the Ag/BiV_0.9925_Mo_0.0075_O_4_ composite systems under visible light irradiation

According to the discussion above, a possible photocatalytic mechanism of Ag/BiV_0.9925_Mo_0.0075_O_4_ was illustrated in Fig. 13. The dopant Mo could effectively enhance the visible light absorption of the BiVO_4_ photocatalyst. Ag/BiV_0.9925_Mo_0.0075_O_4_ composite photocatalysts were irradiated under visible light, and the photoelectrons in the valence band of BiVO_4_ could effectively jump to the conduction band to generate electron–hole pairs. The metallic Ag could accept the electrons, which then recombine with the photogenerated holes and enhance the transfer to the surface of the composite photocatalysts, resulting in the improvement of the separation of electrons and holes. The electrons could react to the O_2_ and transform to •O^2−^. The holes of BiV_0.9925_Mo_0.0075_O_4_ could react with the adsorbed H_2_O molecules and transform to •OH. Meanwhile, the h^+^ could effectively react with the RhB, generating degraded products.

## Conclusions

Herein, a simple hydrothermal synthesis procedure at almost neutral pH conditions and using ammonium carbonate as the structure-directing agent is reported for the preparation of Mo-doped BiVO_4_ powders. Metallic Ag nanoparticles were then deposited on the (040) crystal facet of BiV_0.9925_Mo_0.0075_O_4_. Thus, a photocatalytic system has been successfully constructed by means of the reduction reaction. These synthesis conditions have been shown to significantly influence the increase in the size of the (040) crystallographic facet, as confirmed by XRD and STEM analyses. The XRD indicated that the peak splitting observed at 30.5° is a result of the (040) facets. Ag nanoparticles deposited on the (040) facets can also be seen from the STEM. Furthermore, Ag/BiV_0.9925_Mo_0.0075_O_4_ showed a highly efficient photocatalytic performance for RhB degradation under visible light irradiation. This work could offer new inspiration for the rational utilization of BiVO_4_ photocatalysts with high photocatalytic activity and their applications in the fields of energy production and environmental protection.
